# Do patients perceive a link between a fragility fracture and osteoporosis?

**DOI:** 10.1186/1471-2474-9-38

**Published:** 2008-03-21

**Authors:** Lora Giangregorio, Alexandra Papaioannou, Lehana Thabane, Justin deBeer, Ann Cranney, Lisa Dolovich, Anthony Adili, Jonathan D Adachi

**Affiliations:** 1 Department of Kinesiology, University of Waterloo, Waterloo, Ontario, Canada; 2 Toronto Rehabilitation Institute, Toronto, Ontario, Canada; 3 Department of Kinesiology, McMaster University, Hamilton, Ontario, Canada; 4 Department of Medicine, McMaster University, Hamilton, Ontario, Canada; 5 Department of Surgery, McMaster University, Hamilton, Ontario, Canada; 6 Ottawa Health Research Institute, Clinical Epidemiology Program, Ottawa, Ontario, Canada; 7 Department of Clinical Epidemiology and Biostatistics, McMaster University, Hamilton, Ontario, Canada; 8 Centre for Evaluation of Medicines, St Joseph's Healthcare, Hamilton, Ontario, Canada

## Abstract

**Background:**

To evaluate factors associated with whether patients associate their fracture with future fracture risk.

**Methods:**

Fragility fracture patients participated in a telephone interview. Unadjusted odds ratios (OR, [95% CI]) were calculated to identify factors associated with whether patients associate their fracture with increased fracture risk or osteoporosis. Predictors identified in univariate analysis were entered into multivariable logistic regression models.

**Results:**

127 fragility fracture patients (82% female) participated in the study, mean (SD) age 67.5 (12.7) years. An osteoporosis diagnosis was reported in 56 (44%) participants, but only 17% thought their fracture was related to osteoporosis. Less than 50% perceived themselves at increased risk of fracture. The odds of an individual perceiving themselves at increased risk for fracture were higher for those that reported a diagnosis of osteoporosis (OR 22.91 [95%CI 7.45;70.44], p < 0.001), but the odds decreased with increasing age (0.95 [0.91;0.99], p<0.009). The only variable significantly associated with the perception that the fracture was related to osteoporosis was self-reported osteoporosis diagnosis (39.83 [8.15;194.71], p<0.001).

**Conclusion:**

Many fragility fracture patients do not associate their fracture with osteoporosis. It is crucial for physicians to communicate to patients that an osteoporosis diagnosis, increasing age or a fragility fracture increases the risk for future fracture.

## Background

Osteoporosis has been described as a silent disease until an individual experiences a fragility fracture. A fragility fracture is a fracture that occurs with minimal trauma, such as a fall from a standing height or less. In Canada, age-adjusted incidence rates for hip fracture in 1993–4 were 479 per 100 000 for women and 187 per 100 000 for men, and have been projected to increase four-fold by 2041, and the annual cost of care were estimated at $650 million dollars in 2001 [1,2]. Not only do fragility fractures impose an economic burden, but also there are human costs; fragility fractures can reduce quality of life, increase fear of falling, and often result in impaired mobility and a loss of independence [3-5]. Therapeutic options can reduce the number of new vertebral compression fractures by 40–60% within the first year in individuals with a fracture [6]. For example, the relative risk of fracture associated with alendronate use compared with placebo in women with osteoporosis is 0.47 (95% confidence intervals 0.26–0.79) for hip fractures, 0.52 (0.42–0.66) for radiographic vertebral fractures and 0.70 (0.59–0.82) for all clinical fractures [7]. Increasing age and a history of fragility fracture are independent predictors of subsequent fracture [8]. Therefore, it is essential that individuals who experience fragility fracture be assessed and treated for osteoporosis. However, individuals over the age of 40 years with fragility fracture are not receiving appropriate osteoporosis management [9,10].

If patients do not link their fractures with having a disease, and are not aware that they can actively participate in the disease evolution, then the opportunity to prevent future fractures may be lost [11]. In-depth interviews reveal that many patients with fragility fracture had not associated their fracture with bone fragility. Instead they attributed their fractures to external factors, such as a fall, or slipping on ice [12-14]. In fact, for some patients the belief that their fracture was an accident was so strong that even subsequent fractures were attributed to external situations rather than bone fragility. A survey of peri- and postmenopausal women revealed that although the majority of women (89%) perceived osteoporosis a serious condition, only 29% thought they might be susceptible, and they were less concerned about osteoporosis when compared to cancer, even though the health consequences of osteoporosis are at least equal to that of breast cancer [12,13,15]. With a chronic disease such as osteoporosis, patient self-management is an important component to effective long-term management, especially with self-care issues such as adequate calcium and vitamin D intakes, fall prevention and exercise [16]. There is also a need for long-term adherence to prescribed therapies and self-management. Therefore, it is essential that patients understand that having a fragility fracture increases their risk for subsequent fractures, and that preventative action may be necessary.

Although previous studies have evaluated whether peri- and postmenopausal women and women with fractures perceive their fracture to be related to osteoporosis [12,13,15], it is not known whether fragility fracture patients understand that they are at increased risk of future fracture. Further, no study has evaluated factors, such as age, gender or diagnosis of osteoporosis that may be associated with perceived susceptibility to subsequent fractures in fragility fracture patients. The purpose of the current study was to evaluate whether patients with a fragility fracture perceive themselves to be at risk of future fracture, and to identify factors associated with whether a fragility fracture patient perceives themselves to be at risk of future fractures.

## Methods

### Participants

**Figure 1:**
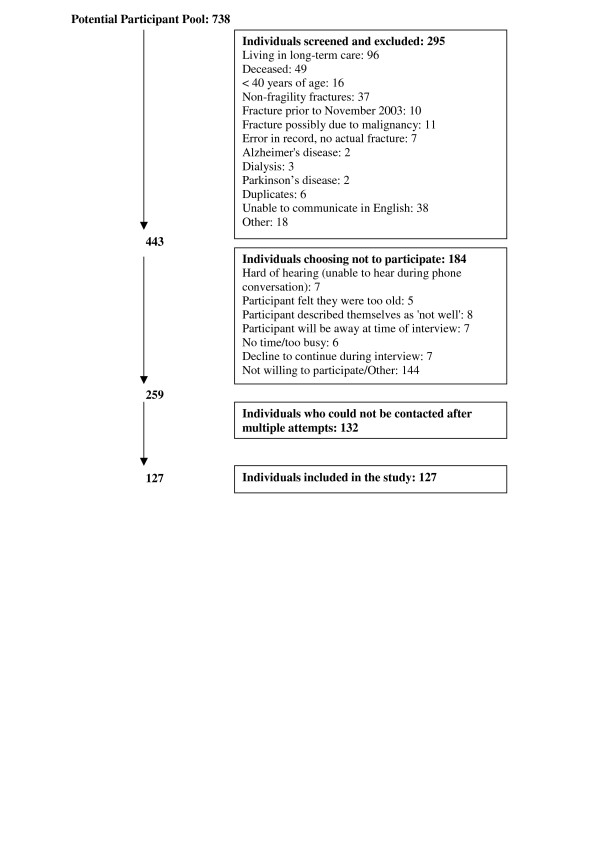
Flow diagram of participant recruitment and inclusion/exclusion.

Patients treated for a fracture by orthopaedic surgeons at two major teaching hospital fracture clinics were contacted. Fractures had to occur at the radius, humerus, femur, rib, tibia (in females only), pelvis or vertebrae to be considered a possible osteoporotic fracture [17]. All patients who had had a fracture at specified sites 2 years prior were identified by orthopaedic surgeons' records, and contacted by the study team. Participants were considered eligible for inclusion in the study if they were 40 years of age or older and if they had experienced a fragility fracture within 18 months of the interview date. Interviews were conducted from September 2005 to June 2006. Exclusion criteria were as follows: fractures of the hands, feet, skull, clavicle, ankle or the tibia in males; fracture due to malignancy; non-fragility fractures; not living in the community; not able to communicate in English; on dialysis; unable to complete interview due to memory loss, dementia or other medical reasons; fracture > 2 years prior to avoid problems with recall (Figure 1). Patients were also asked to describe the incident in which the fracture occurred to verify whether the fracture was a fragility fracture. The study received approval by the Hamilton Health Sciences Research Ethics Board and the St. Joseph's Healthcare Research Ethics Board.

### Survey

A structured telephone interview was conducted with individuals who agreed to participate using a survey instrument developed and pilot-tested for this study by the research team. The interview contained questions about the following: socio-demographic information; prescription medication use in the past year (including treatments for osteoporosis, calcium and vitamin D supplementation); medical and fracture history; family history of osteoporosis; participation in osteoporosis preventative activities (e.g. exercise, intake of calcium rich foods, fall prevention); and participant perceptions about their risk of fracture.

To determine whether individuals who had had a fragility fracture understood what osteoporosis was, whether they associated their fractures with osteoporosis or perceived themselves at risk for future fracture, the following questions were included in the survey: 1) Do you think that breaking your (insert site of fracture here) means that you are at risk for breaking a bone in the future?; 2) Do you think your fracture was related to osteoporosis?; 3) Do you know what osteoporosis is?; and 4) *If yes to question 3: *What do you think it is?. Possible responses to questions 1–3 were "yes", "no" and "unsure". Question 4 was included to confirm whether their understanding of osteoporosis was accurate, and responses were qualitative in nature.

### Statistical Analyses

Descriptive statistics for demographic and clinical participant characteristics are presented as mean (standard deviation [SD]) for continuous variables or count (percent) for categorical variables. The sample size was based on building a stable multivariable model. Simulation studies demonstrate that logistic models require 10 to 15 events per predictor to produce stable estimates [18,19]. We hypothesized that 40% of respondents would associate their current fracture with the potential for future fractures. Therefore, 125 respondents would be required to fit a model with 3–5 predictors. There were no missing data.

For the questions pertaining to perceptions of risk (questions 1 and 3 above), two separate analyses were performed; 1) the response "unsure" was coded as "no"; and 2) the response "unsure" was coded as missing. The following demographic and health-related variables were considered as potential correlates of patients' perceptions of risk: age, gender, education, marital status, living arrangements, site of index fracture, history of a previous fracture, family history of fracture at wrist, spine or hip after the age of 40, use of an assistive aid, ever being told they have osteoporosis, being on an osteoporosis medication, having experienced a loss of height and number of medications. Variables were selected for hypothesis-generating purposes.

Unadjusted odds ratios (OR) and 95% confidence intervals were calculated to identify factors associated with whether patients associated their fracture with being at risk of future fracture, or associated their fracture with osteoporosis. To be conservative, predictors found to be statistically significant at alpha = 0.20 in univariate analysis were entered into multivariable logistic regression models to identify factors that are associated with whether patients associate fragility fracture with and increased risk of future fracture, or with osteoporosis. When two variables that exhibited collinearity were both significant in univariate analyses, only one was included, and the one chosen was based on clinical relevance. We examined the residuals to assess model assumptions. Goodness-of-fit for the models was performed using the Hosmer-Lemeshow test [20]. Data were managed and analyzed using SPSS^® ^software (Version 15.0), and all statistical tests were two-sided. The criterion for statistical significance was set at alpha = 0.05.

## Results

The mean (SD) age of the entire cohort approached was 71.8 years (16.3), where 73.3% were female. The response rate for the telephone interview was 29% (Figure 1) with 127 responding. Demographic characteristics of the 127 participants are listed in Table 1. Females represented 82% (106/127) of the sample. The mean (SD) age was 67.5 (12.7) years. A history of a previous fracture after the age of 40 years was reported by 51 (40%) participants, including 16 wrist fractures, 2 hip fractures, 2 pelvis fractures, 2 spine fractures, 6 humerus fractures, 6 ankle fractures and 25 fractures reported at other sites. A diagnosis of osteoporosis was reported in 56 (44%) participants. Among those with an osteoporosis diagnosis, 45 (80%) were diagnosed before the index fracture. Use of at least one medication, not including supplements, for osteoporosis was reported in 54 (43%) individuals.

**Table 1:** Participant Characteristics

Total Number of Participants	**127**
**Age in years (SD)**	67.5 (12.7)
**Number of Females: n (%)**	106 (82)
**Menopausal Status (females): n (%)**	
Pre-menopausal	4 (3.1)
Peri-menopausal	3 (2.3)
Post-menopausal	95 (74.8)
Unsure	3 (2.3)
**Site of Index Fracture: n (%)**	
wrist	72 (56.7)
hip	26 (20.5)
other femur fracture	10 (7.9)
humerus	5 (3.9)
spine	1 (0.8)
pelvis	1 (0.8)
elbow	12 (9.4)
**Cause of fracture: n (%)**	
Fall from standing height	75 (59.1)
Twisting	3 (2.4)
Slipping on ice	37 (29.1)
Ice skating	3 (2.4)
Re-fracture	1 (0.8)
Fall from standing height while running	2 (1.6)
Hit it on something	2 (1.6)
Unsure	1 (0.8)
Spontaneous	3 (2.4)
**Average time post-fracture in months (SD)**	11.2 (6.3)
**History of previous fracture: n (%)**	51 (40.2)
**Family history of fracture: n (%)**	28 (21.7)
**Self-reported height loss: n (%)**	67 (51.9)
At least one fall in the past year*: n (%)	103 (81.1)
**3 or more falls in the past year: n (%)**	13 (10.1)
**Use assistive aid for mobility (cane, crutches, walker, wheelchair): n (%)**	45 (35.4)
**4 or more prescription medications: n (%)**	53 (41.1)
**Average number of prescription medications: Mean (SD)**	3.7 (3.3)
**Marital Status: n (%)**	
**Single**	7 (5.4)
**Married**	67 (52.8)
**Widowed**	34 (26.4)
**Divorced/Separated**	19 (14.7)
**Living arrangements: n (%)**	
**Live alone**	45 (34.9)
**Live with others**	82 (64.6)

*if the respondent's fracture was due to a fall, this fall was included in the total

### Perceptions of osteoporosis and fracture risk

Over 40% of individuals who had suffered a fragility fracture perceived themselves to be at increased risk of future fracture (Table 2). Despite the fact that 44% of respondents had been told that they have osteoporosis, only 17% of respondents thought their fracture was related to osteoporosis (Table 2), 54% of respondents did not think their fracture was related to osteoporosis and a relatively large proportion of respondents were unsure whether their fracture was related to osteoporosis (29%). Among those that thought their fracture was related to osteoporosis, almost all (20/22) had been told they have osteoporosis. Most of the sample (91%, or n = 115) reported that they knew what osteoporosis was. Among respondents who indicated that they did know what osteoporosis was, the responses were as follows: 95 (75%) respondents reported that it resulted in bone loss or calcium loss, weakening/thinning of the bones or bones becoming porous, 6 (5%) respondents reported only that it was a disease of the bones, 4 (3%) respondents reported that it was crippling or resulted in a crooked spine but did not refer to a disease of bones, 5 (4%) respondents gave an incorrect response but it was related to bones (e.g. loss of bone marrow), 5 (4%) respondents gave an answer related to arthritis or other definition unrelated to osteoporosis or bones. The remaining 12 (9%) respondents reported that they did not know what osteoporosis was or were unsure.

**Table 2:** Responses of individuals who have had a fragility fracture to questions about their perceptions of osteoporosis and fracture risk (n = 127)

	Response: Number of Respondents (%)		
	
	**YES**	**NO**	**UNSURE**
Do you think that breaking your (insert fracture site) means that you are at increased risk of breaking a bone in the future?	55 (43.3)	53 (41.7)	19 (15)
Do you think your fracture was related to osteoporosis?	22 (17.3)	68 (53.5)	37 (29.1)
Have you ever been told that you have osteoporosis?	56 (44.1)	67 (52.8)	4 (3.1)
Do you know what osteoporosis is?	115 (90.6)	11 (8.7)	1 (0.8)

### Logistic Regression Analysis Results

In multivariable analyses, certain variables were significantly related to an individual perceiving that having a fracture meant that they were at increased risk of future fracture (Table 3). The odds of an individual responding "yes" to the question "Do you think that breaking your (wrist, hip, etc) means that you are at increased risk for breaking a bone in the future?" were higher for those that reported a diagnosis of osteoporosis (OR 22.91 [95%CI 7.45;70.44], p < 0.001), but the odds decreased with increasing age (0.95 [0.91;0.99], p < 0.009). When the response "unsure" was coded as "no", the odds ratios associated with a "yes" response associated with having a diagnosis of osteoporosis and increasing age were 14.92 (5.63;39.58) p < 0.001, and 0.94 (0.91;0.98), p < 0.003, respectively. For the question "Do you think that your fracture was related to osteoporosis?" the only variable that remained significantly related to a "yes" response in multivariable analyses was if the individual had ever been told they have osteoporosis (39.83 [8.15;194.71], p < 0.001).

**Table 3:** Estimated odds ratios (95% CIs) for variables associated with perceiving that a fragility fracture results in an increased risk of future fracture, or that the fragility fracture is related to osteoporosis.

Question	Variable (reference)	OR (95% C.I.)	p-value
Do you think that breaking your (insert fracture site) means that you are at increased risk of breaking a bone in the future? (unsure coded as missing, n = 108)	Ever told you have osteoporosis (No)	22.91 (7.45; 70.44)	0.001
	Age (per year)	0.95 (0.91;0.99)	0.009
	Sex (male)	1.41 (0.332;6.00)	0.640
Do you think that breaking your (insert fracture site) means that you are at increased risk of breaking a bone in the future? (unsure coded as no, n = 127)	Ever told you have osteoporosis (No)	14.93 (5.63;39.58)	0.001
	Age (per year)	0.94 (0.91;0.98)	0.003
	Sex (male)	2.23 (0.58;8.57)	0.244
Do you think that your fracture was related to osteoporosis? (unsure coded as missing, n = 90)	Ever told you have osteoporosis (No)	39.83 (8.15;194.71)	0.001
	Use an assistive aid (No)	2.43 (0.65;9.03)	0.185
Do you think that your fracture was related to osteoporosis? (unsure coded as no, n = 127)	Ever told you have osteoporosis (No)	18.10 (3.98;82.27)	0.001
	Use an assistive aid (No)	1.52 (0.54;4.25)	0.426

## Discussion

The current study demonstrates that a substantial number of individuals who suffer a fragility fracture do not perceive themselves to be at increased risk for future fracture, and an even greater number of individuals do not associate their fracture with osteoporosis. The odds that individuals perceived themselves at risk for future fracture were higher among individuals who had been told that they had osteoporosis, but decreased with increasing age. If individuals with fragility fracture do not perceive themselves at risk for future fracture, they may be less likely to accept or adhere to recommended treatment [11,21], which may partially explain the reports of poor management among this patient group. Recent systematic reviews have demonstrated that in Canada and internationally, individuals who suffer fragility fractures are not receiving adequate osteoporosis management [9,10].

Perceived susceptibility to osteoporosis has been evaluated among community-based women over the age of 40 who had not been diagnosed with osteoporosis; the majority of women perceive their risk to be lower than other women their age, and they attributed their lower risk primarily to their own preventative behaviours [22]. The current study is unique because it evaluated perceived risk of fracture among community-living individuals who had recently suffered a fragility fracture and are therefore at higher risk of future fracture than individuals who have not had a fracture. However, even among this higher risk group, many individuals with a prior fragility fracture still do not perceive an increased susceptibility to future fracture. Perceived risk of future fracture decreased with increasing age among fragility fracture patients, which is alarming considering that increasing age and a history of fracture are both independent predictors of subsequent fracture [8]. Further, it has been demonstrated that older individuals with fragility fracture are not prescribed osteoporosis treatment as often as younger individuals [9]. A negative relationship between perceived risk of chronic diseases (i.e. osteoporosis, breast cancer and heart disease) and age has been demonstrated previously among community-living individuals [23,24]. It has been suggested that women may perceive themselves to be less susceptible to osteoporosis with age because the longer they live without experiencing the disease, the more distant the threat of the disease appears, the more likely they are to perceive the disease occurrence to be low, or the more dissimilar they view themselves to women with the disease [24]. However, the participants in the current study have already experienced a consequence of osteoporosis, namely, a fragility fracture. It appears many individuals do not recognize their fracture is an indicator that they are at risk for osteoporosis and future fractures. Therefore, it is crucial to communicate to individuals who have suffered a fragility fracture that they may have osteoporosis and are at increased risk of future fractures [8].

The likelihood that a patient with a fragility fracture perceived themselves to be at increased risk of future fracture was much greater if they had been told that they had osteoporosis. However, the clear communication of an osteoporosis diagnosis to fragility fracture patients is inconsistent, and there is a care gap between the occurrence of a fragility fracture and osteoporosis diagnosis and treatment [9,10,25]. Interestingly, the current study demonstrated that only about one in five individuals thought their fracture was related to osteoporosis. This is particularly concerning considering that almost half of the sample had been given a diagnosis of osteoporosis, and 80% of those were diagnosed *before *the index fracture. The results of the current study suggest that patients may not understand that osteoporosis leads to an increased risk of future fracture, that there is a lack of understanding of what osteoporosis is, or that patients do not associate having a fragility fracture with having a chronic disease, namely osteoporosis.

A recent study demonstrated that perceived susceptibility to osteoporosis was related to current use of antiresorptive medication [11]. Similarly, that having a previous bone density test or diagnosis of osteoporosis were the only factors associated with active consideration of or current use of osteoporosis medication in hip fracture patients [21]. Therefore, communicating to patients that having a fragility fracture increases their susceptibility to future fracture may be crucial for increasing the probability that patients will accept osteoporosis therapy and participate in chronic disease management. It has been demonstrated that if women had an understanding of the impact of a self-management behaviour (i.e. calcium intake) related to osteoporosis they were more likely to act on that knowledge [26]. Self-management is an important component of chronic disease management; a recent meta-analysis revealed that self-management programs for diabetes mellitus and hypertension produced statistically and clinically significant benefits [27]. A patient-centred approach targeting the patient's perception of future fracture risk may be a critical element in improving osteoporosis management. In a prospective study, providing women with a decision aid about benefits and risks of hormone therapy, educational tools and feedback about densitometry resulted in increased use of osteoporosis medications, including calcium and vitamin D [28]. A recent quasi-experimental non-randomized trial demonstrated that patient education in addition to informing the physician of the fracture and providing treatment guidelines significantly improved both osteoporosis investigation and treatment rates, resulted in improved persistence with therapy and was cost-saving [29,30]. Further, the method of communication may be crucial. For example, a recent study demonstrated that patients preferred simple bar charts with absolute lifetime risk depicted on them [31].

The current study has a few limitations. Among the potential pool of participants, 42% chose not to participate and 30% could not be contacted to determine if they were eligible. We do not have bone density data to confirm whether all participants have osteoporosis. However, having a fragility fracture is a predictor of future fractures independent of bone density [8]. We report history of prior fracture after the age of 40, but we did not ascertain whether prior fractures were also fragility fractures. The proportion of respondents reporting a diagnosis of osteoporosis is relatively high compared to previous reports of the proportion of fragility fracture patients diagnosed with osteoporosis after fracture [9,10], suggesting that we may have over-represented individuals with diagnosed osteoporosis. However, even among our sample there were a substantial number of individuals who did not make the link between their fragility fracture and osteoporosis. Given the observed relationship between having an osteoporosis diagnosis and perception of future fracture risk in the current sample, inclusion of a more representative sample may have actually increased the strength of our findings.

## Conclusion

In summary, many individuals who suffer fragility fracture do not associate their fracture with osteoporosis. Communicating a diagnosis of osteoporosis to the patient was strongly related to perceptions of future fracture risk, as was age. It is crucial for health care providers to communicate to the patient that increasing age and having a fragility fracture increases the risk for future fracture, so that patients can become active participants in chronic disease management. It may also be necessary to rethink the way we convey messages about fracture risk to patients; rather than relying on brief verbal communications between patient and physician during follow-up visits, the message could be emphasized using well designed written materials that include attention-grabbing graphics. As well the message that fragility fracture equals risk of future fragility fracture needs to become part of a nationwide post-fracture care initiative that targets patients in hospital so that all patients are made aware of the risk. Future research should investigate whether risk perceptions or a diagnosis of osteoporosis influences acceptance of or adherence to recommended therapies, or the impact of patient-centred educational interventions on the patient's perception of risk.

## Competing interests

J.D. Adachi

I have been involved in clinical trials with the following companies: Amgen, Eli Lilly, Glaxo Smith Kline, Merck Frosst, Novartis, NPS-Allelix, Pfizer, Procter & Gamble, Roche, Sanofi Aventis, Wyeth. I have been a consultant to: Amgen, Astra Zeneca, Eli Lilly, Glaxo Smith Kline, Merck Frosst, Novartis, Pfizer, Procter & Gamble, Roche, Sanofi Aventis, Servier, Wyeth.

A. Cranney

I have been involved in clinical trials with Eli Lilly and Zelos Therapeutics and received honoraria for speaking from Novartis, Procter & Gamble and Merck Frosst.

A. Papaioannou

I am or have been a consultant or on the speaker's bureau for the following: Aventis Pharma, Eli Lilly Canada Inc., Merck Frosst Canada, Novartis Pharmaceticals Canada Inc., Proctor and Gamble Pharmaceuticals.

L. Dolovich

I have been a co-investigator on research grants supported by Aventis, Eli Lilly and Merck Frosst.

L. Giangregorio

I have been a co-investigator on a research grant supported by Merck Frosst.

L. Thabane, J. deBeer, A. Adili

Nothing to disclose

## Authors' contributions

LG and AP conceived of the research questions and developed the initial proposal. LG, AP, JDA, LD and AC conceived of the final proposal, including study design, methods and literature review, funded by the Drummond Foundation. LG, AP, JDA, LD, AC and LT developed and pilot tested the telephone survey. JD, AA and LG were responsible for developing and implementing the participant recruitment and screening process, and data acquisition. LG and LT performed data analysis. JD, AA, JDA, AC and AP contributed clinical expertise with respect to survey development, data analysis and data interpretation. LG was primarily responsible for writing the initial draft of the manuscript, but all authors contributed to and approved subsequent revisions.

## Pre-publication history

The pre-publication history for this paper can be accessed here:


